# Screening and diagnosis of hemoglobinopathies in Germany: Current state and future perspectives

**DOI:** 10.1038/s41598-022-13751-8

**Published:** 2022-06-13

**Authors:** Carmen Aramayo-Singelmann, Susan Halimeh, Pia Proske, Abinuja Vignalingarajah, Holger Cario, Morten O. Christensen, Raina Yamamoto, Alexander Röth, Dirk Reinhardt, Hans Christian Reinhardt, Ferras Alashkar

**Affiliations:** 1 Department of Pediatrics III, University Children’s Hospital Essen, University of Duisburg-Essen, Essen, Germany; 2 Coagulation Center Rhein-Ruhr, Duisburg, Germany; 3 Department of Hematology and Stem Cell Transplantation, West German Cancer Center, University Hospital Essen, University of Duisburg-Essen, Essen, Germany; 4 Department of Pediatrics and Adolescent Medicine, University Medical Center Ulm, Ulm, Germany; 5 MVZ Dr. Eberhard and Partner, Dortmund, Germany

**Keywords:** Diseases, Medical research, Molecular medicine

## Abstract

This monocentric study conducted at the Pediatric and Adult Hemoglobinopathy Outpatient Units of the University Hospital of Essen summarizes the results of hemoglobinopathies diagnosed between August 2018 and September 2021, prior to the introduction of a general newborn screening (NBS) for SCD in Germany (October 2021). In total, 339 patients (pts.), 182 pediatric [50.5% males (92/182)] and 157 adult pts. [75.8% females (119/157)] were diagnosed by molecular analysis. The most common (parental) descent among affected pts. were the Middle Eastern and North African/Turkey (Turkey: 19.8%, Syria: 11.8%, and Iraq: 5.9%), and the sub-Saharan African region (21.3%). Median age at diagnosis in pediatric carriers [N = 157; 54.1% males (85/157)] was 6.2 yrs. (range 1 (months) mos.–17.8 yrs.) and 31 yrs. (range 18–65 yrs.) in adults [N = 53; 75.2% females (115/153)]. Median age at diagnosis of homozygous or compound-heterozygous disease in pediatric pts. (72% (18/25) females) was 3.7 yrs., range 4 mos.–17 yrs. (HbSS (N = 13): 2.5 yrs., range 5 mos.–7.8 yrs.; HbS/C disease (N = 5): 8 yrs., range 1–8 yrs.; homozygous/compound heterozygous β-thalassemia (N = 5): 8 yrs., range 3–13 yrs.), in contrast to HbH disease (N = 5): 18 yrs. (median), range 12–40 yrs. Hemoglobinopathies represent a relevant health problem in Germany due to immigration and late diagnosis of second/third generation migrants. SCD-NBS will accelerate diagnosis and might result in reduction of disease-associated morbidity. However, diagnosis of carriers and/or disease-states (i.e. thalassemic syndromes) in newly immigrated and undiagnosed patients will further be delayed. A first major step has been taken, but further steps are required.

## Introduction

Hemoglobinopathies encompass genetic disorders affecting either the production (i.e., quantitative disorders, characterized by a defective globin production) or structure (i.e., qualitative or functional disorders) of the hemoglobin (Hb) protein^1^. The two main representatives are thalassemic syndromes and sickle cell disease (SCD). Precise epidemiological data concerning the prevalence and the number of affected children born each year in Germany can only be estimated vaguely as hemoglobinopathies are rare diseases in Germany with the main distribution areas extending from sub-Saharan Africa to the Mediterranean, the Near and Middle East, Southeast Asia, and the Indian subcontinent^2,3^. In 2012, estimates suggested that in Germany the number of carriers of hemoglobinopathy genes amounted to 400,000 people, resulting in an estimated prevalence rate of 0.5 to 1% of the overall population (5% of the immigrant population)^1,4^. For SCD an estimated birth prevalence of 1:5000–1:7500 for Germany is currently being assumed. These numbers are based on a supportive publication analyzing health insurance data and data collected in four pilot studies conducted in metropolitan areas in Germany past 2012^5^. Data addressing the birth prevalence of thalassemic syndromes in Germany, however, are less clear. Due to an increase in migration from high prevalence countries within the past years (yrs.), more children affected by hemoglobinopathies are expected to be born within the course of the next decades^6^. This in summary highlights the importance of premarital and/or antenatal counseling at hemoglobinopathy reference centers in the presence of an already confirmed hemoglobinopathy, including heterozygous disease states, or in patients originating from endemic countries, if a hemoglobinopathy/carriership is highly probable, as early diagnosis and treatment in disease states remains crucial and is associated with a delay of potential disease-associated morbidity and mortality rates in these patients, which is far too often disregarded^7–11^.

## Materials and methods

### Study design and participants

This is a single-center, retrospective study carried out at the Pediatric and Adult Hemoglobinopathy Outpatient Units (PAHOs) of the University Hospital Essen in Germany summarizing all initial diagnoses of heterozygous, compound heterozygous and homozygous hemoglobinopathies [thalassemic syndromes and structural hemoglobin variants (abnormal hemoglobins)] identified between August 2018 and September 2021, prior to implementation of newborn screening (NBS) for SCD in Germany. All patients were referred to the PAHOs by health professionals (HPs) for evaluation and genetic testing in the context of suspicion of a hemoglobinopathy due to patients’ ancestry with additional evidence of microcytic, hypochromic anemia. After obtaining written and informed consent, genetic, laboratory, and clinical data, including age at diagnosis, gender, ethnic heritage/country of origin/birth were documented. Patients with already confirmed diagnoses before referral to the PAHOs were excluded. The study was approved by the Ethics Committee of the University of Duisburg-Essen and conducted in accordance with the Declaration of Helsinki (21-10344-BO).

### Methods

In minors or patients unable to give consent, genetic testing was performed after obtaining written informed consent from parents and/or guardians. Following capillary electrophoresis (CE), diagnoses were confirmed according to international standards via molecular globin gene genetic analyses by polymerase chain reaction (PCR), sequencing and/or multiplex ligation-dependent probe amplification (MLPA). In pediatric patients, molecular analyses were carried out at the Coagulation Center Rhein-Ruhr, Duisburg, and at the Hemoglobin Laboratory of the University Hospital Ulm. In adult patients, molecular analyses were performed at the Medical Care Center Dr. Eberhard & Partner Dortmund, Dortmund, and at the Coagulation Center Rhein-Ruhr, Duisburg.

### Iron status

Serum ferritin concentrations (SF) (µg/L) for the assessment of iron status were evaluated and age-adjusted according to the World Health Organization (WHO) guidelines (2020) for determination of iron deficiency^12^.

### Ethical approval

The study involving human participants was reviewed and approved for retrospective analysis and use of data was approved by the Ethical Committee of the Faculty of Medicine at the University Hospital of Duisburg-Essen (21-10344-BO). The study was conducted in accordance with the 1964 Helsinki Declaration. Written patients' informed consent was obtained from all patients or their parent or legal guardian in the case of children under 18 years of age. The patients/participants/parents/legal guardians provided their written informed consent to participate in this study.

## Results

In a total of 339 patients, 182 pediatric [50.5% males (92/182)] and 157 adult patients [75.8% females (119/157)], a hemoglobinopathy was confirmed by molecular analysis. The most common countries of origin among affected patients were the Middle Eastern and North African (MENA) (35.1%), especially Syria (11.8%) and Iraq (5.9), the Eastern European and Central Asian (24.6%), particularly Turkey (19.8%), and the sub-Saharan African region (21.3%) (Table 1).

**Table 1:** Migration background among patients with hemoglobinopathies diagnosed at the Pediatric and Adult Hemoglobinopathy Outpatient Units (PAHOs) of the University Hospital Essen between August 2018 to September 2021 (N = 339).

Geographic region (N; %)	Country of origin	N (%)
Middle East and North Africa (MENA) (119; 35.1)	Syria	40 (11.8)
	Iraq	20 (5.9)
	Lebanon	12 (3.5)
	Morocco	12 (3.5)
	Egypt	10 (2.9)
	Iran	8 (2.4)
	Palestine	7 (2.1)
	Algeria	5 (1.5)
	Saudi Arabia	3 (1.5)
	Jordan	1 (0.3)
	Yemen	1 (0.3)
East Europe and Central Asia (83; 24.6)	Turkey	67 (19.8)
	Kosovo	7 (2.1)
	Bulgaria	4 (0.9)
	Afghanistan	3 (0.9)
	Kazakhstan	1 (0.3)
	Yugoslavia	1 (0.3)
Sub-Saharan Africa (72; 21.3)	Nigeria	20 (5.9)
	Ghana	13 (3.8)
	Kongo	12 (3.5)
	Guinea	8 (2.4)
	Angola	5 (1.5)
	Cameroon	5 (1.5)
	Togo	3 (0.9)
	Ivory Coast	2 (0.6)
	Kenia	1 (0.3)
	Senegal	1 (0.3)
	Sierra Leone	1 (0.3)
	Uganda	1 (0.3)
European Countries (21; 6.2)	Italy	11 (3.2)
	Greece	6 (1.8)
	Germany	2 (0.6)
	Poland	1 (0.3)
	Romania	1 (0.3)
South Asia (15; 4.4)	India	10 (2.9)
	Pakistan	4 (1.1)
	Sri Lanka	1 (0.3)
(East) Asia and Pacific (10; 2.9)	Indonesia	4 (1.1)
	Thailand	2 (0.6)
	China	2 (0.6)
	Philippines	1 (0.3)
	Vietnam	1 (0.3)
South America and Caribbean (8; 2.4)	Brazil	7 (2.1)
	Dominican Republic	1 (0.3)
NV (11; 3.2)		11 (3.2)

*MENA* Middle East and North Africa, *NV* no value.

### Hemoglobinopathy carriers

**Figure 1:**
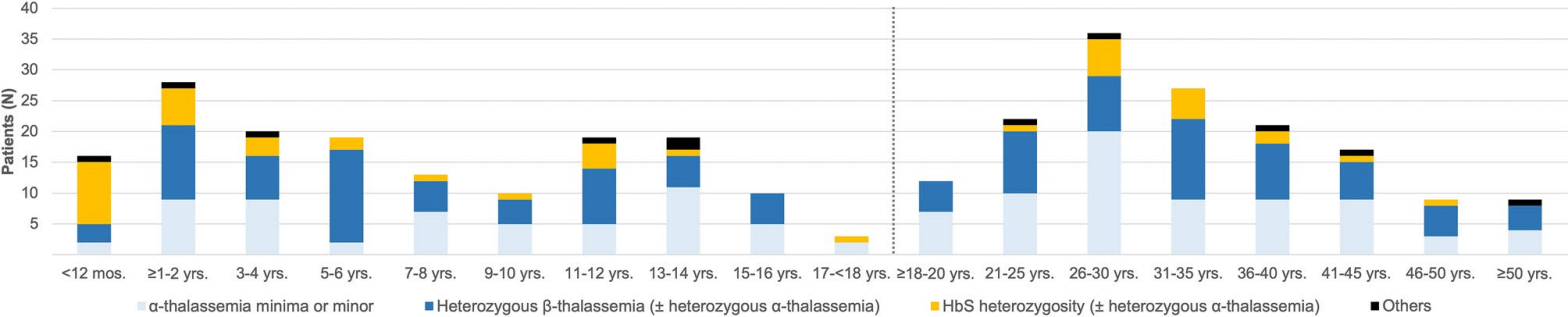
Age distribution of hemoglobinopathy carries identified at the Pediatric and Adult Hemoglobinopathy Outpatient Units (PAHOs) of the University Hospital Essen (Aug. 2018–Sept. 2021) (N = 310). Others (heterozygosity): α-thalassemia/Hb G-Philadelphia (N = 1), HbC (N = 2), HbD (N = 1), HbE (N = 1), Hb Presbyterian (N = 1), HbS/Hb Q-Iran (N = 1); delt-beta (δβ)-thalassemia (β-thalassemia) (N = 2), hereditary persistence of fetal hemoglobin (HPFH) (N = 1); mos., months; yrs., years.

Median age at diagnosis in pediatric patients was 6.2 yrs. (range 1 (months) mos.–17.8 yrs.) [N = 157; 54.1% males (85/157)] and 31 yrs. (range 18–65) in adults [N = 153; 75% females (115/153)]. The age distribution at the time of diagnosis in these patients is presented in Fig. 1.

#### Neonates to adolescents

The age of diagnosis was within the first yr. of life in 10.2% (16/157) of children and < 5 yrs. of age in 40.8% (64/157) of patients. In newborns of parents or in siblings affected by SCD [homozygosity for HbS (N = 10) or compound heterozygous HbS/D disease (N = 1)] (11/16), prior CE revealed a median HbS fraction of 20% (range 5–37; N = 10). In children heterozygous for HbS (± heterozygous α-thalassemia and/or Hb Q-Iran), 79% (20/29) of diagnoses were made within the first five yrs. of life [65% males (13/20)]: Hb concentration (median): 11.6 g/dl, range: 9–13.6 (N = 19); mean corpuscular volume (MCV) (median): 77.6 fl, range 58.8–94.4 (N = 19); mean corpuscular hemoglobin (MCH) (median): 25.6 pg, range 16.4–33 (N = 19); HbS (CE, %): 32%, range 5–40 (N = 19). In 14 out of 15 (93%) of these children in whom the country of birth was known, were born in Germany. In children ≥ 6 to < 18 yrs. of age and confirmation of heterozygosity for HbS [31% (9/29) (median); CE, %)] ± heterozygous α-thalassemia, the country of birth was known in five patients, of whom three (60%) were born in Germany. In these three patients, despite a positive family history for SCD, molecular testing was carried out by the age of 12 yrs. (HbS: 33% (median) CE, %).

In children with (± compound) heterozygous α- and/or heterozygous β-thalassemia (N = 125), median age of diagnosis was 7.8 yrs., range 3 mos.–17.3 yrs. [53% males (66/125)]. Of them, 4% (5/125) were diagnosed within the first yr. of life [Hb (median): 10.5 g/dl, range 9–14; MCV (median): 69.6 fl, range 51.7–75, MCH (median): 21.3 pg, range 16.6–75)], 42% (52/125) within the first 5 yrs. of life [Hb (median): 10.8 g/dl, range 3.9 (additional evidence of iron deficiency: SF: 2 μg/l)–16.2 (N = 51); MCV (median): 62 fl, range 48.6–80.5 (N = 51); MCH (median): 19.4 pg, range 11.6–26.2 (N = 51)], respectively. Reasons for molecular testing were due to a positive family history with affected siblings, including transfusion-dependent β-thalassemia (TDT), SCD, and/or thalassemia carrier states (siblings or parents) [34.4% (11/32)]. However, most of these patients were referred for genetic testing by local pediatrics in the context of a highly suspected hemoglobinopathy due to abnormal blood count [65.6% (21/32)].

#### Adults

In adult patients with regard to age of diagnosis, a consistently wide age range (up to ≥ 50 yrs. of age) was observed. The highest prevalence for diagnosis was observed in the age group > 26 to 30 yrs. (23.5%, N = 36). After the age of 30 yrs., there was a regredient, but still clearly increased rate of first diagnoses up to the 45th yr. of life (Fig. 1). By the age of 30 yrs., 45.8% (N = 70) of the total number of adult patients were diagnosed, 63.4% (N = 97) by the age of 35 yrs., respectively. Overall median Hb in adult patients by the date of diagnosis was 11.5 g/dl, range 4.3–15.9 (N = 149) [MCV (median): 66 fl, range 53.4–86.9 (N = 149); MCH (median): 21.1 pg, range 15.8–29.1)]. Of note, exact data concerning the place of birth or the date of entry into Germany were available in 37 adult patients [age at diagnosis (median): 33 yrs., range 19–65; 56.8% females (21/37)]. Of them, 24.3% (9/37) were born in Germany, 13.5% (5/37) entered Germany within the 5th yr. of life. In total, 56.8% (21/37) of these patients, including second/third generation migrants born in Germany, have immigrated to Germany with their parents by the age of 18 yrs. All patients were referred to the APHAs by health professionals (HPs) for further evaluation and genetic testing in the context of suspicion of a hemoglobinopathy due to patients’ ancestry with additional evidence of microcytic, hypochromic anemia.

In Table 2, clinical data of pediatric and adult hemoglobinopathy carriers [excluding: heterozygosity for HbC (N = 3), HbD (N = 1), HbE (N = 1), Hb Presbyterian (N = 1), hereditary persistence of fetal hemoglobin (HPFH) (N = 1)] diagnosed at the PAHAs of the University Hospital Essen are summarized.

**Table 2:** Clinical data of pediatric and adult hemoglobinopathy carriers (N = 303).

	Pediatric patients (N = 154)			Adult patients (N = 149)		
	HbS heterozygosity [± heterozygous α-thalassemia; ± Hb Q-Iran (N = 1)]	α-thalassemia minima and/or minor (± Hb G-Philadelphia (N = 1)	Heterozygous β-thalassemia [± heterozygous α-thalassemia; heterozygous δβ-thalassemia (N = 1)]	HbS heterozygosity (± heterozygous α-thalassemia)	α-thalassemia minima or minor	Heterozygous β-thalassemia [± heterozygous α-thalassemia; ± heterozygous δβ-thalassemia (N = 1)]
Number	29	59	66	16	70	63
Gender (female, %)	10 (34)	28 (47)	31 (47)	12 (75)	59 (82)	42 (66)
Age at diagnosis (mos./yrs.) (median, range)	28 mos., 1–213 mos.	108 mos., 3–207 mos.	70 mos., 3–199 mos.	32 yrs., 24–49 yrs.	29 yrs., 18–65 yrs.	32 yrs., 18–65 yrs.
Hb (g/dl) (median, range)	11.8, 9–15.2 (N = 26)	11.8, 3.9–78.5	10.8, 6.5–15.4	12.1, 6.9–15.4 (N = 13)	11.6, 4.3–15.5 (N = 69)	11,3, 8–15.9
MCV (fl) (median, range)	79.9, 58.8–94.6 (N = 26)	68.9, 48.6–84.9 (N = 57)	60.3, 51.7–80.5	77.1, 57.9–86.9 (N = 13)	71.9, 53.4–84.1 (N = 69)	62.8, 55–79.4
MCH (pg) (median, range)	25.7, 16.4–33 (N = 26)	21.7, 11.6–27.3 (N = 57)	18.8, 14–27.4	25.6, 16.4–29.1 (N = 13)	22.8, 15.8–27.9 (N = 69)	20, 17.1–27.1
ARC (nl) (median, range)	50.3, 24.2–169.8 (N = 17)	42.4, 14.7–184.9 (N = 14)	71, 18–120.5 (N = 38)	67.6, 57.1–100 (N = 6)	63.5, 25.2–347.4 (N = 13)	102.6 37.3–159.2 (N = 20)
LDH (U/l) (median, range)	279, 233–548 (N = 16)	200, 169–394 (N = 15)	257, 149–416 (N = 38)	229.5, 199–327 (N = 6)	210, 158–284 (N = 13)	187, 152–325 (N = 20)
Total bilirubin (mg/dl) (median, range)	0.4, 0.2–0.7 (N = 15)	0.5, 0.2–2.4 (N = 14)	0.5, 0.2–2.5 (N = 38)	0.5, 0.4–1.1 (N = 6)	0.5, 0.2–2.3 (N = 13)	0.7, 0.2–5.8 (N = 20)
Ferritin (μg/l) (median, range)	21, 4–118 (N = 21)	19, 2–129 (N = 53)	21, 3–153 (N = 64)	22, 2–314 (13)	27, 2–547 (N = 66)	54, 4–527 (N = 60)

*ARC* absolute reticulocyte count, *Hb* hemoglobin, *HPFH* hereditary persistence of fetal hemoglobin, *LDH* lactate dehydrogenase, *MCH* mean corpuscular hemoglobin, *MCV* mean corpuscular volume, *mos*. months, *NV* no value, *yrs*. years.

### Iron status in adults

In females (N = 155), median overall SF concentration (available in N = 107), irrespective of age and menopausal status, was 23 μg/l, range 2–527 (chronic kidney disease (CKD) and status post recent intravenous iron replacement therapy prior to referral). In adult females, a cut-off SF concentration < 15 μg/l, indicating iron deficiency, was observed in 37.3% of patients (40/107), while in 12.1% (13/107) of the patients, adequate SF concentrations (≥ 100 μg/l) were present. Iron deficiency was more frequently found to be present in females as compared to male patients and was irrespective of specific age groups in females. Even though, the current analysis was not powered to differentiate between the prevalence of iron deficiency according to menopausal status in hemoglobinopathy carriers, as true menopausal status was routinely not obtained (Table 3).

**Table 3:** Serum ferritin concentrations in adult hemoglobinopathy carriers (N = 153).

Adult hemoglobinopathy carriers					
Gender (N)	Age (yrs.) (N; %)	Hb g/dl (median, range) (N)	SF levels (μg/l) (median, range) (N)	SF < 15 μg/l (N; %)	SF (≥ 100 μg/l (N; %)
Females (115)	≥ 18 to < 30 (53; 46)	10.9, 4.3–13.5 (52)	20, 2–527 (51)	21 (41)	6 (11)
	≥ 30 to < 40 (35; 30)	10.9, 7.2–15.5 (33)	26, 2–253 (30)	9 (30)	5 (16)
	≥ 40 to < 50 (23; 20)	11.1, 6.9–13.4 (23)	20, 2–64 (22)	9 (39)	0 (0)
	≥ 50 (4; 4)	10.6, 9.4–11.9 (4)	87, 10–190 (4)	1 (25)	2 (50)
Males (38)	≥ 18 to < 30 (14; 37)	12.9, 8.9–15.9 (14)	121, 47–307 (13)	0 (0)	10 (76)
	≥ 30 to < 40 (12; 32)	13.1, 11–14.9 (10)	100, 6–220 (10)	1 (10)	5 (50)
	≥ 40 to < 50 (7; 18)	13.2, 11.2–15.4 (7)	246, 160–484 (6)	0 (0)	5 (83)
	≥ 50 (5; 13)	13.4, 11.8–14 (5)	169, 56–408 (5)	0 (0)	3 (60)

*Hb* hemoglobin, *SF* serum ferritin, *yrs*. years.

#### Hemoglobinopathies in pediatric and adult siblings

Throughout observation time, molecular testing with subsequent confirmation of a hemoglobinopathy carrier status was performed in 27 families with two (N = 21) or three (N = 6) siblings each.

In pediatric patients, a hemoglobinopathy carrier status in a total of 48 patients out of 21 families was confirmed. Median age at diagnosis was 9 yrs., range 1 mos.–15 yrs., with 14 sibling pairs being tested in a joint presentation due to a positive family history. In seven pediatric patients (six families) molecular testing was delayed, of which two patients of a single family were evaluated within a joint presentation. Median duration in this group of patients following identification to subsequent sibling testing was nine mos., range 2 mos.–2 yrs. For additional clarification, in one offspring of an already identified sibling, diagnosis was confirmed two mos. after birth.

In adults, molecular diagnosis confirming a carrier status was made in 6 families with two sibling pairs each, of which two families were evaluated in a joint presentation due to a positive family history. The median duration to sibling testing in already diagnosed patients (4 families) was 19 months, range 1–33 months.

#### Disease states in pediatric and adult patients

Median age at diagnosis in pediatric patients (72% (18/25) females) was 3.6 yrs., range 4 mos.–17 yrs. Patients’ characteristics at date of diagnosis, regarding the type of hemoglobinopathy present, are presented in Table 4. Reasons for referral of pediatric patients were (a) an unclear microcytic, hypochromic hemolytic anemia, (b) genetic ancestry in terms of the parent’s origin, (c) diseased/affected siblings, (d) disease-specific symptoms (pain crises, femoral head necrosis, and/or need for transfusions), or (e) a combination of two or three of the listed reasons. Fourteen of the 18 pediatric patients (77.8%) with SCD were of sub-Saharan Africa descent (parental ancestry), 22.2% (4/8) from MENA. Three of the affected children were siblings. Of the sickle cell patients in whom the country of birth was known [83.3% (15/18)], 80% (12/15) were born in Germany. Median age at diagnosis in these patients was 3.1 yrs., range 5 mos.–8.3 yrs. In pediatric patients with non-transfusion-dependent β-thalassemia (NTDT) and/or transfusion-dependent β-thalassemia (TDT), parental ancestry was in equal proportions from MENA or Turkey, with an additional patient originating from Eastern Europe. In these patients, the country of birth was known in two patients, however, both were born in Germany (age at diagnosis: 3 and 8 yrs.). Median age at diagnosis in NTDT and/or TDT patients was 8.2 yrs., range 4 mos.–13.9 yrs., respectively.

**Table 4:** Disease states in pediatric and adult patients (N = 28).

	Pediatric patients (N = 25)					Adult patients (N = 3)
	HbS/S (± heterozygous α-thalassemia)	HbS/C disease	β-thalassemia (homozygous β^0^, homozygous β^+^, compound heterozygous β^++^; ± heterozygous α-thalassemia; ± homozygous HPFH)	HbH disease		HbH disease
Number	13	5	5	2		3
Gender (female, %)	8 (61)	5 (100)	4 (80)	2 (100)		3 (100)
Age at diagnosis (mos./yrs.) (median, range)	30 mos., 5–94 mos.	8 yrs., 1–8 yrs.	8 yrs., 3–13 yrs.	12 yrs.	17 yrs.	33 yrs., 18–40 yrs.
Hb (g/dl) (median, range)	10, 7.2–11.3	10.9, 9.7–11	9.7, 8.1–11.6 (transfused)	10.1	10.5	9.1, 7.7–9.6
MCV (fl) (median, range)	78.5, 71.7–94.6	75.3, 60.9–78	68.6, 61.8–79.2 (transfused)	66.7	82.3	83.7, 73.2–103.6
MCH (pg) (median, range)	26, 22.2–30	24.9, 22.9–26.5	20.8, 19.2–27.3 (transfused)	19.6	27.6	25.8, 23.6–27.9
ARC (nl) (median, range)	231.8, 77.5–393.4 (N = 12)	120.6, 119.4–189.6	94.8, 43.3–188.1 (transfused; N = 4)	121.8	NV	NV
LDH (U/l) (median, range)	543, 349–668 (N = 7)	297, 268–365	483, 195–634 (transfused; N = 4)	NV	NV	NV
Total bilirubin (mg/dl) (median, range)	1.6, 0.9–4.5 (N = 7)	1.1, 1–2.3	2.7, 1.5–4.1 (transfused; N = 4)	NV	NV	NV
Ferritin (μg/l) (median, range)	98, 34–372 (N = 8)	70, 28–185	137, 20–201 (transfused)	NV	20.7	94, 45–106

*ARC* absolute reticulocyte count, *Hb* hemoglobin, *HPFH* hereditary persistence of fetal hemoglobin, *LDH* lactate dehydrogenase, *MCH* mean corpuscular hemoglobin, *MCV* mean corpuscular volume, *mos*. months, *NV* no value. *yrs*. years.

For further completeness, in one adult patient the diagnosis of HbS/C-disease in the context of a proliferative retinopathy (stage V: tractional retinal detachment) was made. This patient immigrated to Germany 2 yrs. prior to referral.

Of note, in contrast to other types of hemoglobinopathies, diagnosis of HbH disease in female patients (N = 5) was delayed (median age at diagnosis: 18 yrs., range 12–40) with 60% of patients (3/5) being diagnosed ≥ 18 yrs. of age. Molecular testing in these patients was indicated in the context of (antenatal) hemolytic anemia with or without additional evidence of iron deficiency or in patients immigrating from high-risk countries with or without a positive family history for thalassemia.

## Discussion

As a result of constant immigration of people from endemic countries and due to second and third generation migrants born in Germany, hemoglobinopathies represent a relevant health problem in Germany^4,13,14^. The variety of different clinical manifestations ranges from mild microcytic, hypochromic anemias to anemias associated with high morbidity and mortality rates, if not diagnosed within the first yrs. of life and treated adequately^15–19^.

Immigration had reached a high in the yrs. 2015 (476,649 asylum applicants) and 2016 (745,545 asylum applicants). After rising for nine consecutive yrs. between 2008 and 2016, particularly due to the high influx of asylum seekers, with around 2.1 million inflows and net migration of 1.1 million persons, total migration to Germany declined thereafter over following yrs. Migration, however, also involves emigration, with Germany, the United Kingdom, Spain and France, continue to be the main European destination countries. In the reporting year 2021 to date (Nov 2021), 132,666 initial asylum applications were received by the Federal Office. The most represented high-risk nationalities in 2021 included: Syria [50,218 initial applications (IAs)], Afghanistan (20,454 IAs), Iraq (13,275 IAs), Turkey (6254 IAs), Somalia (3384 IAs), Eritrea (2866 IAs), Iran (2418 IAs), and Nigeria (2349 IAs), etc. 23,865 of these asylum applicants (18.0%) were children under the age of one born in Germany. Excluding these children, a total of 108,801 IAs were submitted^20^.

In 2020, around 21.9 million people with a migration background (immigrants and their descendants) lived in Germany, of which 94.9% of persons with a migration background lived in western Germany and Berlin. Overall, this corresponds to 26.7 percent of the general population. Of these people, 11.5 million have a German passport, around 10.3 million are foreigners, and around 13.6 million were thus born abroad and immigrated. Even though a large proportion of migrants do not necessarily originate from a hemoglobinopathy high-risk country, these numbers, however, do illustrate the current multi-ethnic population in Germany. In 2020, people with a Turkish migration background accounted for the largest share with around 2.75 million people (12.6%) compared to 4.2% of people with an Italian migration background, and on average, people with a migration background in 2020 were 31.3 yrs. old^21^. This is particularly noteworthy since Germany, Turkey, Italy, and Greece have been closely linked by more than fifty years of migration history since the late 1960s and represents a potentially further and important explanation for the high number of first diagnoses in adult patients in our patients. Our data are in line with the observations made in our retrospective analysis and data obtained by Kohne et al., with the overall majority of patients identified were of Turkish descent. In contrast, the proportion of patients with Italian ancestry was significantly lower in our analysis (3.2%)^4^. However, this may be explained by the change in migration over the last years. Even though precise data concerning the age of immigration were not available to state of the files in all patients, 54% of adult patients were diagnosed between the 21st and 35th yr. of life by molecular testing. Most of them were second or even third generations migrants born in Germany, but also included immigrates born outside of Germany, who immigrated < 5 yrs. of age. In this group, heterozygous α-thalassemias, including one HbH patient, were the most frequent thalassemias with about 45.9% (39/85) of cases observed, followed by 35.3% (30/85) of β-thalassemic carriers [HbS heterozygosity: 10.6% (9/85); other hemoglobinopathies (e.g., heterozygosity for HbC) or combination forms: 8.2% (7/85)]. The prevalence in these age groups was dependent of ethnic origin, with 67.5% (56/83) of hemoglobinopathy carriers originating from the MENA(T) region.

Unfortunately, this analysis was not powered to evaluate the number of affected offspring in identified patients or their respective partners. Given a general risk of inheritance of 25–50% if either one or both parents are affected, the implementation of routine partner screening for both, qualitative and quantitative hemoglobin disorders, in affected patients or patients migrating from high-risk countries, including their offspring, in terms of prevention to minimize the risk of disease states (e.g., HbS/β-thalassemia) and patient education, at the latest within the course of prenatal planning, which is far too often disregarded, is becoming of increasingly importance in Germany. This statement is based on the fact that, in contrast to countries with a high prevalence, such as Italy, Greece, and Turkey, national hemoglobinopathy screening programs have been successfully implemented for several years^22–25^. The need for implementation of screening programs in countries with rising patient numbers with no national screening programs such is further reflected by the high number of diagnosed siblings in the present analysis, in both pediatric and adult patients, throughout observation time. If intrafamilial testing was not desired by one patient at the time of diagnosis, it was subsequently recommended.

Of considerable importance was the fact, that in a high number of adult females (37.2%), irrespective of age and menopausal status, the diagnosis of a concomitant and in some cases long-standing and thus, not sufficiently treated iron deficiency anemia was made. Consequently, in the event of high-grade suspicion for a hemoglobinopathy, irrespective of the presence of a concurrent iron deficiency anemia, patients should be offered appropriate diagnostics despite the associated costs that molecular genetic testing might entails, as in particular α-thalassemia can only be ruled out by genetic testing^26^.

Since 2021 screening for SCD is part of the Extended Newborn Screening (ENS) in Germany, with screening for SCD being reimbursed by the statutory health insurance via the uniform assessment standard (“Einheitlicher Bewertungsmaßstab”, EBM) as announced by the Federal Joint Committee (“Gemeinsamer Bundesausschuss”, G-BA)^27,28^. Screening for SCD will be carried out using tandem mass spectrometry (MS/MS), high-performance liquid chromatography (HPLC) or CE, followed by confirmation via molecular diagnosis, if SCD is highly suspected^27^. Of note, genetic testing in suspected carrier states or newly immigrated patients will not be covered by the NBS. The aim of newborn screening for SCD is to enable early diagnosis and treatment of infants and young children born in Germany to reduce morbidity and mortality rates associated with the SCD. Consequently, in the long term, improvement in the training of parents and relatives could be assumed. Prior to implementation of NBS for SCD, data from the General Local Health Insurance Fund (“Allgemeine Ortskrankenkasse”, AOK) revealed that in only 15.4% of cases, children born in 2009 and 2010, were identified within the first or second quarter of life, with a median duration up the seventh quarter, which was further supported in our group of pediatric patients, with a median age of 2.5 yrs. in HbSS and 8 yrs. of age in HbS/C patients, respectively.

In summary, a major step in the right direction has been taken, however, further steps must follow. It is gratifying to note that the general awareness for hemoglobinopathies has improved significantly over the past yrs., since the largest proportion of patients included in this analysis were referred to our PAHAs by attentive HPs, both pediatricians and general practitioners, for confirmation of a suspected hemoglobinopathy.

## Data Availability

All datasets generated for this study are included in the article/supplementary material.

## Abbreviations

AOK: “Allgemeine Ortskrankenkasse”/General Local Health Insurance Fund
ARC: Absolute reticulocyte count
CE: Capillary electrophoresis
CKD: Chronic kidney disease
EBM: “Einheitlicher Bewertungsmaßstab”/uniform assessment standard
ENS: Extended Newborn Screening
G-BA: “Gemeinsamer Bundesausschuss”/Federal Joint Committee
Hb: Hemoglobin concentration
HPs: Health professionals
HPFH: Hereditary persistence of fetal hemoglobin
HPLC: High-performance liquid chromatography
IA: Initial applications
LDH: Lactate dehydrogenase
MCH: Mean corpuscular hemoglobin
MCV: Mean corpuscular volume
MENA(T): Middle Eastern and North African (Turkey)
MLPA: Multiplex ligation-dependent probe amplification
mos: Months
MS/MS: Tandem mass spectrometry
NS: Newborn screening
NTDT: Non-transfusion-dependent β-thalassemia
NV: No value
PAHOs: Pediatric and Adult Hemoglobinopathy Outpatient Units
PCR: Polymerase chain reaction
SCD: Sickle cell disease
SF: Serum ferritin concentrations
TDT: Transfusion-dependent β-thalassemia
WHO: World Health Organization
yrs: Years
